# From aggregate structure to disease trajectory: the role of the proteostasis network

**DOI:** 10.1098/rstb.2025.0276

**Published:** 2026-10-01

**Authors:** Jessica Tittelmeier, Carmen Nussbaum-Krammer

**Affiliations:** Chair of Neuroanatomy, Institute of Anatomy, LMU Medizin, Ludwig-Maximilians-Universität München, Munich, Germany

**Keywords:** protein aggregation, structural polymorphs, neurodegenerative diseases, prion-like propagation, proteostasis network, protein quality control, chaperones, protein disaggregation

## Abstract

Neurodegenerative diseases are characterized by the accumulation of a small number of misfolded proteins, yet they differ markedly in cellular vulnerability and clinical outcome. We propose that this heterogeneity arises from the interaction between two largely orthogonal variables: the structural identities of protein aggregates and the identities of the cellular proteostasis networks (PNs) that engage them. Protein aggregates exist as structurally diverse assemblies with distinct intrinsic properties. At the same time, PNs vary between cell types, disease states and ageing in their composition, capacity and plasticity. As a result, the same aggregate can be efficiently cleared in one cellular context but persist, be remodelled, or give rise to self-propagating species in another. Likewise, the same PN can be protective or harmful, depending on the aggregate it encounters. Because neither aggregates nor PNs are static, their interaction evolves over time, leading to dynamic feedback between aggregate identity and PN state that shapes disease onset, progression and clinical symptoms. This view reframes neurodegenerative disease as a failure of compatibility between specific aggregate structures and the cellular protein quality control network that processes them rather than as a uniform collapse of proteostasis, and provides a framework for understanding selective vulnerability and disease trajectories.

This article is part of the Theo Murphy meeting issue ‘ProteostaSys: a systems view of proteostasis’.

## 1. Introduction

Neurodegenerative diseases associated with the accumulation of misfolded proteins display remarkable clinical and pathological heterogeneity [1–3]. Even when linked to aggregation of the same protein, such as tau or *α*-synuclein, these diseases can differ markedly by anatomical distribution of pathology, routes of intercellular spreading, progression rate and clinical presentation [1,4,5]. Understanding how such diverse disease manifestations emerge from a limited set of aggregation-prone proteins remains a central challenge in the field.

A major conceptual advance in addressing this problem has been the prion strain concept. According to this principle, a single protein sequence can adopt multiple self-propagating conformations with distinct biological properties [6–9]. Early support for this idea came from yeast studies showing that identical Sup35 sequences can give rise to heritable [PSI⁺] variants—the prion form of Sup35—with distinct phenotypes [10]. Crucially, recent cryo-electron microscopy studies have provided direct structural evidence for this concept in human neurodegenerative diseases: distinct amyloid structures of the same protein have now been isolated from patients with different clinical syndromes [11–13]. Disease-associated amyloid assemblies therefore are not singular pathological entities but exist as structurally distinct fibril polymorphs, further diversified by alternative splicing and post-translational modifications. These distinct ‘aggregate identities' differ in molecular architecture, packing density, surface chemistry, membrane interactions and dynamic features such as fragmentation and seeding. As a result, aggregates formed by the same protein can encode distinct biological behaviours.

At the same time, aggregated proteins encounter cellular environments that are far from uniform. The molecular machinery responsible for maintaining protein homeostasis (proteostasis), collectively referred to as the cellular protein quality control (PQC) or proteostasis network (PN), varies substantially across tissues, cell types and cellular states [14–16]. This variability extends beyond quantitative differences in expression levels to qualitative differences in network organization, including the relative contribution of specific chaperone systems, ubiquitin ligases or autophagy adaptors. We refer to these cell- and state-specific PN configurations as ‘PN identities’, which determine how misfolded or aggregated proteins are recognized, remodelled or degraded.

Together, these observations define two largely orthogonal axes of heterogeneity that shape neurodegenerative disease outcomes: heterogeneity in the structural identities of protein aggregates and heterogeneity in the cellular PQC networks (figure 1). An aggregate may be efficiently cleared in one cellular context, but persist, damage organelles and self-amplify in another. Likewise, a given PN may handle some aggregates effectively while failing against others. This framework offers a unifying explanation for selective vulnerability, divergent disease trajectories and distinct spreading behaviours. Importantly, it shifts the focus from whether proteostasis fails to which PN fails against which aggregate, and at which step—recognition, remodelling or degradation.

**Figure 1.**
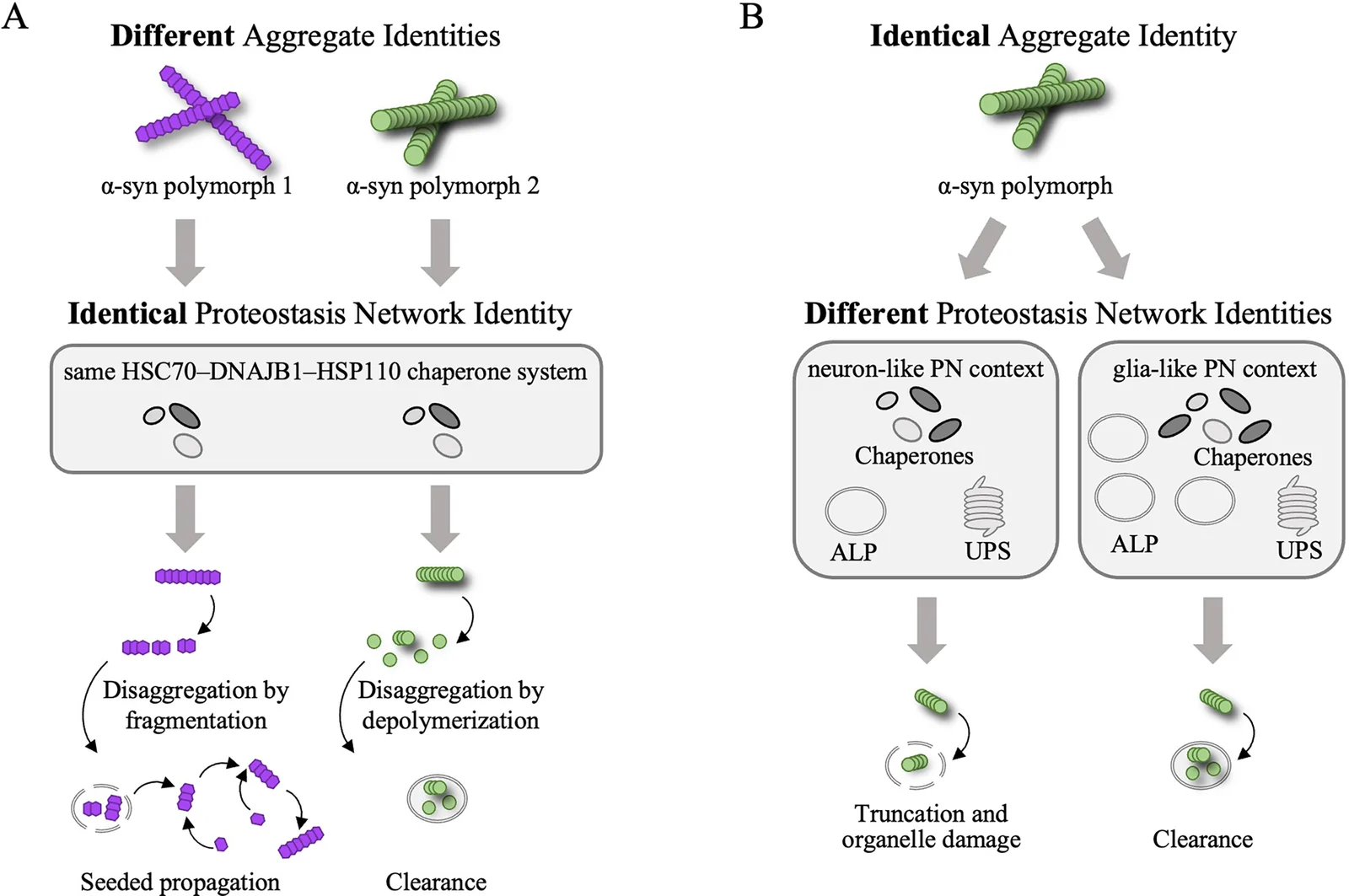
Aggregate–PN compatibility determines cellular outcomes. (A) Distinct aggregate identities can be processed differently within the same proteostasis network (PN) context. This is illustrated here using two *α*-synuclein polymorphs engaged by the same HSC70–DNAJB1–HSP110 chaperone system, which can promote either fragmentation or depolymerization and thereby lead to different downstream outcomes, including seeded propagation or clearance [17]. (B) Conversely, the same aggregate identity can be processed differently in distinct PN contexts. This is illustrated here using the same *α*-synuclein aggregate encountering different cellular proteostasis environments, resulting in divergent outcomes such as clearance or truncation-associated persistence and organelle damage [18]. Together, these examples illustrate that cellular outcome depends on the compatibility between aggregate identity and PN identity. ALP, autophagy–lysosome pathway; UPS, ubiquitin–proteasome system.

In the following sections, we first examine the structural diversity of aggregate identities, then explore how PNs differ across cell types and states, and finally discuss how specific combinations of aggregate and PN identities give rise to distinct disease manifestations.

## 2. Aggregate identities: structural heterogeneity

Protein aggregation is often described as a transition from soluble to insoluble states. However, a single gene product can assemble into a diverse landscape of aggregate identities that differ in molecular architecture, properties and dynamic behaviour. These differences define distinct aggregate identities, which represent the first axis of heterogeneity shaping neurodegenerative disease outcomes.

### (a) Sources of structural variability

One fundamental source of aggregate heterogeneity is that proteins can populate different assembly states, ranging from monomers to soluble oligomers, protofibril intermediates and mature fibrils, each with distinct biochemical and functional characteristics. A second source is structural polymorphism: even assemblies formed from an identical primary amino acid sequence can adopt alternative fibrillar architectures within a given aggregate state [6,7,9]. The conceptual basis for same-sequence conformational diversity emerged from studies on mammalian prions, where fibrillar assemblies of the prion protein (PrP) were associated with distinct disease phenotypes despite sharing an identical primary sequence [19,20]. This principle was subsequently demonstrated experimentally in yeast, where recombinant Sup35 yeast prion protein assembles into structurally distinct amyloid conformations without any sequence alteration [21,22]. More recently, advances in structural biology have extended these findings to animal prion models, showing that prion strain diversity can reflect distinct PrP amyloid architectures formed from identical sequences *in vivo* [23–25].

Same-sequence structural polymorphism is now well established for other disease-associated amyloid proteins. Recombinant *α*-synuclein assembles into multiple fibrillar polymorphs from an identical sequence, differing in protofilament organization, packing and surface properties [26]. These structural differences are associated with distinct mechanical characteristics and stability, as shown by nanomechanical measurements and cryo-electron microscopy of recombinant fibrils assembled *in vitro* [11,27].

Beyond same-sequence polymorphism, aggregate identities can also arise from sequence or chemical variation. In animal prion models, closely related but non-identical PrP sequences, such as hamster 263K and mouse RML strains, form distinct amyloid architectures *in vivo* [13]. Alternative splicing can generate protein isoforms with different domain compositions and aggregation-prone regions, thereby altering which sequence segments participate in fibril core formation. In tau, differential inclusion of exon 10 produces 3-repeat and 4-repeat isoforms that assemble into distinct filament architectures *in vitro* and in disease [28–30]. Proteolytic truncations similarly reshape the accessible folding landscape by removing sequence elements that constrain fibril assembly, giving rise to structurally distinct aggregates [31,32]. Post-translational modifications (PTMs) further diversify aggregate identities by modifying the chemical properties of the polypeptide chain. A recent large-scale proteomics study identified 145 PTMs and 195 cleavage sites of tau in different tauopathies [33]. Phosphorylation of *α*-synuclein has been shown to promote fibril formation *in vitro* [34], while in tau it can stabilize fibrillization [35].

Together, same-sequence polymorphism and aggregation of sequence- or chemistry-modified variants define the spectrum of aggregate identities accessible to a given gene product.

### (b) Intrinsic biophysical and biochemical properties of aggregate identities

Structural differences arising from the sources outlined above translate into distinct intrinsic biophysical and biochemical properties that can be quantified *in vitro*, independent of cellular context. These properties define aggregate identities at a molecular level and constrain how assemblies behave, once formed.

Differences in molecular packing, protofilament organization and fold architecture determine intrinsic stability and rigidity. In mammalian prion systems, PrP assemblies associated with distinct prion isolates exhibit characteristic resistance to proteolysis and denaturation despite identical primary sequence, reflecting differences in conformational stability [20,36]. Similar observations were made in yeast, where alternative Sup35 amyloid conformations assembled from identical sequences display markedly different resistance to chemical denaturation and susceptibility to mechanical fragmentation [21,22].

Recombinant *α*-synuclein polymorphs formed from the same sequence differ in fibril width, stiffness and nanomechanical properties, as measured by atomic force microscopy [27]. Cryo-electron microscopy revealed that these differences arise from distinct protofilament folds and inter-protofilament interfaces [11]. Similarly, recombinant tau filaments assembled from different isoforms or under different conditions adopt distinct core architectures, resulting in differences in fibril organization and surface topology [29,37]. Aggregate identities further differ in surface properties, as different folds expose different sets of charged, polar and hydrophobic residues, resulting in characteristic surface chemistries [11,29]. Finally, aggregates exhibit distinct dynamic behaviours, including fragmentation propensity and the generation of seeding-competent species, which were first quantified in yeast prions and later observed for *α*-synuclein and tau *in vitro* [26,38–40].

Taken together, polymorphs of identical sequences and aggregates formed from sequence- or chemistry-modified variants give rise to assemblies with distinct intrinsic physical and biochemical phenotypes. These properties define what an aggregate identity is in molecular terms and set the stage for how aggregates are subsequently handled by cellular PQC networks.

## 3. Proteostasis network identities: composition, sources of variability and functional capacity

### (a) Core components and network organization

Cellular proteostasis is maintained by an interconnected PQC network, also referred to as the PN. This network comprises modules that act at distinct stages of the protein life cycle, including co-translational folding, post-translational remodelling, sequestration or holding of non-native proteins and targeted clearance. Core components include ATP-dependent chaperone systems such as Hsp70 with J-domain proteins (JDPs) and nucleotide exchange factors (NEFs), Hsp90 with a diverse set of co-chaperones and ATP-independent small heat shock proteins (sHSPs) that act as holdases [41,42]. Protein degradation is mediated primarily by the ubiquitin–proteasome system (UPS) and the autophagy–lysosome pathway (ALP), supported by specialized receptors and adaptors that control cargo selection and delivery [43].

Crucially, these components do not operate as independent pathways. Instead, molecular chaperones, degradation systems and trafficking machinery form a highly interconnected and partially redundant network that is tightly coupled to protein synthesis and turnover. PN capacity therefore emerges from the architecture, connectivity and regulation of the network as a whole, not from the activity of individual components in isolation.

We refer to these integrated, network-level properties as PN identities, emphasizing that they represent stable yet adaptable configurations of the cellular PQC machinery. As with aggregate identities, PN identities define how a cell engages and processes misfolded or aggregated proteins.

### (b) Sources of variability in PN identity

PN identities vary widely across cellular contexts. One major source of variability is cell type and differentiation state [44]. Different cell types deploy distinct baseline PQC programmes that reflect their physiological demands. In the nervous system, for example, single-cell transcriptomic analyses predict neuron-class-specific PN configurations, with systematic differences between excitatory neurons, inhibitory neurons and non-neuronal cells [14]. Such cell-type-specific PN identities probably contribute to selective vulnerability in neurodegenerative diseases, where aggregation-prone proteins are often broadly expressed but pathology manifests in highly specific cell populations [45]. PN identity may differ between subcellular compartments and organelles, which can expose the same aggregate to distinct proteostasis components and quality control pathways [46]. PN identity also varies across the life course. Developmental transitions reshape folding demand and quality control requirements to support specialized cellular functions, whereas ageing is associated with a progressive decline in PQC capacity, narrowing the range of protein states that can be effectively managed [47]. Metabolic state and nutrient signalling provide an additional layer of regulation. Nutrient availability and energy status influence protein synthesis rates, degradation capacity and autophagic flux, thereby shifting the relative balance between folding, maintenance and clearance pathways according to cellular demands [48]. Finally, PN identities are shaped by stress history. Transient, sublethal stress can in some contexts induce long-lasting increases in stress resistance and PN capacity, a phenomenon often referred to as hormesis [49–51]. However, repetitive, chronic, or poorly resolved stress can progressively erode PN capacity and bias engagement toward maladaptive pathways.

### (c) Functional capacity of proteostasis network identities

Differences in PN composition and organization translate into differences in functional capacity, which determines not only whether aberrant protein states are detected, but also how they are routed and processed over time.

A first dimension of functional capacity includes recognition and engagement. In the Hsp70 system, the diversity, localization and relative abundance of JDPs strongly influence substrate selection [52]. Other co-chaperones such as Hop (Hsp70/Hsp90-organizing protein) coordinate substrate transfer between Hsp70 and Hsp90 [53]. Recognition also occurs at clearance interfaces, where specialized receptors (e.g. Rpn10 and Rpn13) and adaptors (e.g. p62) govern entry into the UPS or ALP, respectively [54,55].

A second dimension is routing and processing. Hsp70 NEFs bias substrate fate: Hsp110 family NEFs enhance disaggregation activity, while theBcl-2-associated athanogene (BAG) family NEFs link Hsp70 to distinct downstream pathways, with BAG1 directing clients to the UPS and BAG3 favouring autophagy-based clearance [56]. Another example is the regulatory proteasome subunit PSMD11/Rpn-6, which enhances proteasome activity and proteotoxic stress resistance in stem cells and *Caenorhabditis elegans* [57,58].

A third dimension is plasticity. An important feature of the cellular PN is the ability to expand or reconfigure capacity in response to stress. Some networks show robust inducibility through specific stress responses, whereas others operate near their functional limits. Hormetic conditioning can shift the PN toward more protective configurations [49–51]. Conversely, age-associated decline of PN capacity and attenuation of stress responses reduce plasticity and increase vulnerability to proteotoxic challenges [59,60].

Together, these dimensions define how PN identities differ in their capacity to accommodate or fail against specific aggregate identities.

## 4. Interactions between aggregate identities and PN identities

Sections 2 and 3 introduced aggregate identities and PN identities as independent sources of heterogeneity. In this section, we examine how their interaction gives rise to distinct cellular outcomes. We first consider how different aggregate identities are handled within the same PN context, and then how the same aggregate identity can produce divergent outcomes when processed by distinct PN identities.

### (a) Distinct aggregate identities are processed differently within the same PN context

We focus on amyloid fibril fragmentation as a well-studied example of how aggregate identity shapes engagement by the PN. Fragmentation changes the number of growth-competent fibril ends and therefore directly influences aggregate propagation. As a result, chaperone-mediated disaggregation can either promote or suppress propagation, depending on whether processing primarily generates new fibril ends or instead leads to progressive dissolution of the fibrils [21,61–63].

In yeast, protein disaggregation is mediated by the ring-forming molecular chaperone Hsp104, a member of the AAA+ protein family [64]. Hsp104-driven fragmentation of Sup35 amyloid fibrils is essential for inheritance of the [PSI⁺] prion state [65]. Within an otherwise constant cellular context, different Sup35 amyloid conformers formed from the same protein sequence are propagated with markedly different efficiencies [21]. These conformers differ in intrinsic fibril brittleness and division rates, correlating with large differences in seed number and strain phenotype [21]. Sup35 conformers with higher intrinsic stability are less efficiently fragmented and consequently propagate less robustly [21]. Subsequent work demonstrated that the balance between fibril growth and fragmentation determines prion strain behaviour, establishing aggregate structure as a key determinant of biological outcome [38].

Direct evidence of this principle was obtained using a minimal reconstituted chaperone system consisting of Hsp104, Hsp70 (Ssa1) and Hsp40 (Sis1) acting on defined Sup35 conformers [66]. Distinct conformers exhibited different modes of disaggregation: one is preferentially fragmented, generating new seeds, whereas another undergoes dissolution [66]. Thus, this system provides a direct experimental example of aggregate–PN compatibility, in which a constant PN produces distinct biological outcomes depending solely on aggregate identity.

In metazoan cells, which lack Hsp104, protein disaggregation is mediated by the Hsp70 system, primarily HSC70 together with its co-chaperones, the JDP DNAJB1 and an HSP110-type NEF [41,67,68]. The efficiency of this machinery depends strongly on aggregate type [69].

In the context of metazoan disease, *α*-synuclein provides a particularly clear example of how aggregate-intrinsic features alone bias processing. Distinct *α*-synuclein fibril polymorphs assembled from identical monomers under different buffer conditions exhibit divergent persistence and seeding behaviour in the same cellular background [17,26,67,70]. Using a reconstituted Hsp70 disaggregation system *in vitro*, these *α*-synuclein fibrils are actively remodelled, but with efficiencies that vary across polymorphs [17]. Notably, distinct fibril types follow different processing routes: some undergo gradual depolymerization from fibril ends, whereas others are fragmented into shorter fibrils and oligomeric species [17]. These differences correlate with intrinsic fibril fragility and with differential binding of HSC70 and DNAJB1, and translate into distinct seeding efficiencies in the same biosensor cell line [17]. Importantly, these experiments were performed in a defined and constant chaperone environment, demonstrating that aggregate-intrinsic properties alone are sufficient to bias processing outcomes, thereby driving divergent downstream effects such as seeding efficiency.

Fibril modifications further modulate these interactions. C-terminally truncated *α*-synuclein assemblies show reduced engagement with the Hsp70 disaggregation machinery, consistent with diminished accessibility of chaperone binding sites in this region [17]. Conversely, phosphorylation of serine 129 within the C-terminus—the most prevalent PTM in Lewy bodies—was shown to enhance DNAJB1 binding [71]. Although the consequences of this modification for disaggregation efficiency remain to be determined, these findings suggest that disease-associated PTMs may directly modulate PN-mediated processing, with potential implications for disease manifestation and progression.

Tau fibrils show comparable behaviour. Distinct polymorphs derived from different tau isoforms are disassembled with markedly different efficiencies by the Hsp70 disaggregation system, suggesting that polymorph-specific processing can translate into differences in propagation efficiency even when the PN is unchanged [72].

Taken together, these examples illustrate that aggregate identity strongly biases engagement and processing by a given chaperone machinery (figure 1A). In the next section, we consider the converse situation: how changes in PN identity can redirect the fate of an otherwise identical aggregate.

### (b) The same aggregate identity is processed differently in distinct PN contexts

Changes in PN identity can profoundly alter the fate of a given aggregate. In yeast, this principle is mechanistically well established. Both inhibition and overexpression of Hsp104 eliminate the [PSI⁺] prion state despite continued amyloid formation, demonstrating that altered PN parameters alone are sufficient to shift aggregate fate from propagation to loss [17,65–73]. Modulation of other chaperone components has comparable effects, as changes in the cytosolic Hsp70 system, including Ssa and Ssb, influence de novo [PSI⁺] formation and reshape the distribution and stability of prion variants [74].

Evidence that PN context influences prion properties in metazoans initially came from mammalian prion transmission studies. Upon transmission to a new host, a specific prion strain may be faithfully propagated or mutate to a new strain, resulting in altered phenotypic properties, a phenomenon described as strain adaptation [75–77]. A similar form of context-dependent adaptation has recently been demonstrated for *α*-synuclein: a defined *α*-synuclein strain isolated from multiple system atrophy (MSA) patients alters its strain properties after passage in mice [78].

One model proposed to explain such behaviour suggests that prions do not consist of a single discrete conformer, but rather of a dynamic population of structurally related assemblies maintained under host-specific selective pressure, consistent with the quasi-species concept [8,79–81]. Within a given host environment, host-specific factors preferentially amplify those conformers best adapted to the local cellular context. Upon transmission to a new host, a different subset of conformers from this ensemble may be selectively amplified, resulting in a shift in dominant structures and consequently in phenotypic properties [8,79–81]. An important implication of this selection model is that the newly dominant conformer was already present within the original ensemble, albeit at low abundance.

An alternative, not mutually exclusive, model goes beyond selective amplification of pre-existing conformers and instead emphasizes a PN-mediated processing model, in which interactions with the host PN actively generate conformers characteristic of a given prion population. In this view, aggregate–PN interactions do not merely select among pre-existing assemblies, but they actively reshape aggregate identity through differential processing, including truncation, incomplete disassembly, or other PN-dependent remodelling events. These PN-generated conformers may then be preferentially propagated within the host. Upon transfer to a new host, engagement with a different PN environment could again actively remodel the conformer ensemble, leading to the emergence and subsequent propagation of new dominant species with distinct structural and phenotypic properties.

Increasing evidence indicates that host PN context contributes to strain selection and phenotypic outcome in *α*-synuclein pathology, although the underlying mechanisms remain incompletely understood. At the tissue level, analysis of MSA and Lewy body disease reveals that differences in cellular composition and cytopathology correlate with distinct *α*-synuclein seeding patterns [82]. At the cellular level, factors present in oligodendrocytes—but not neurons—can convert identical *α*-synuclein fibrils into a highly potent glial cytoplasmic inclusion-like strain with enhanced seeding and propagation capacity, indicating that specific intracellular milieus can impart distinct pathological *α*-synuclein strain properties [83]. In a separate experimental context, using different cellular models and *α*-synuclein fibril preparations, neurons and glial cells were found to process internalized *α*-synuclein fibrils differently: neurons preferentially accumulate truncated, degradation-resistant species, whereas glial cells more efficiently clear the same fibrils [18]. Such differences in intracellular processing are probably to influence downstream pathological outcomes. Accumulation of *α*-synuclein in lysosomes can promote vesicle rupture, allowing the aggregates to escape into the cytosol and seed further aggregation [84–87]. Moreover, inefficient lysosomal degradation may lead to rerouting of aggregates toward secretion, thereby enhancing intercellular spreading [88–91]. Thus, PN identity influences not only whether aggregates are cleared or persist, but also whether they traffic between cells.

Direct manipulation of PN activity further illustrates context-dependent aggregate handling. Modulation of proteostasis capacity in cells differentially affects the processing and seeding dynamics of defined *α*-synuclein polymorphs [70]. Even more directly, engineered variants of the yeast disaggregase Hsp104 can acquire the ability to process amyloid substrates that are not handled by the wild-type enzyme, including *α*-synuclein, demonstrating that qualitative changes in PN activity can expand or restrict the range of aggregates that can be productively engaged [92].

Together, these studies show that aggregate fate is not determined by aggregate properties or PN capacity alone, but emerges from their interaction (figure 1B). Aggregate identity biases how a given PN engages and processes an aggregate, while changes in PN identity can redirect the fate of an otherwise identical aggregate toward clearance or propagation. These reciprocal effects establish aggregate–PN compatibility as a key determinant of cellular outcomes.

This framework helps explain why apparently conflicting results have been reported in the literature. For example, some studies report that *α*-synuclein fibrils are mainly depolymerized by the Hsp70 disaggregation machinery without the accumulation of harmful intermediate species [62,89], whereas others describe efficient fragmentation into shorter, more seeding-competent assemblies [61,68]. Similarly, studies focusing on the same cell type can reach different conclusions: in some experimental settings, passage of *α*-synuclein fibrils through oligodendrocytes—but not neurons—converts them into species with enhanced seeding and propagation capacity [83], whereas in other contexts oligodendrocytes clear *α*-synuclein fibrils more efficiently than neurons [18]. These observations are often interpreted as contradictory. However, they are probably all correct and instead reflect differences in the intrinsic properties of the fibrils used and in the cellular proteostasis networks engaged in each study. Because multiple fibril structures are possible and proteostasis networks are highly plastic, divergent processing outcomes are expected rather than surprising.

## 5. Conclusion

Heterogeneity in neurodegenerative disease cannot be fully explained by aggregate properties or proteostasis failure in isolation. Instead, disease onset, progression and clinical manifestation reflect the compatibility between aggregate identities and PN identities. Aggregate structure defines intrinsic physical and biochemical properties, whereas PN composition and activity determine how these properties are interpreted and processed within specific cellular contexts.

Importantly, neither aggregates nor PNs are static. Aggregate identities evolve through processes such as truncations, PTMs and selective amplification of specific fibril structures. In parallel, PN identities are reshaped by ageing, stress history and increasing pathological burden. The resulting co-evolution of aggregates and PNs (figure 2) provides a systems-level explanation for disease trajectories, selective vulnerability and transitions between pathological states. In this context, neurons may be particularly vulnerable to such mismatches between aggregate identity and PN identity because they lack dilution through cell division and must maintain proteostasis throughout their often exceptionally long lifetime.

**Figure 2.**
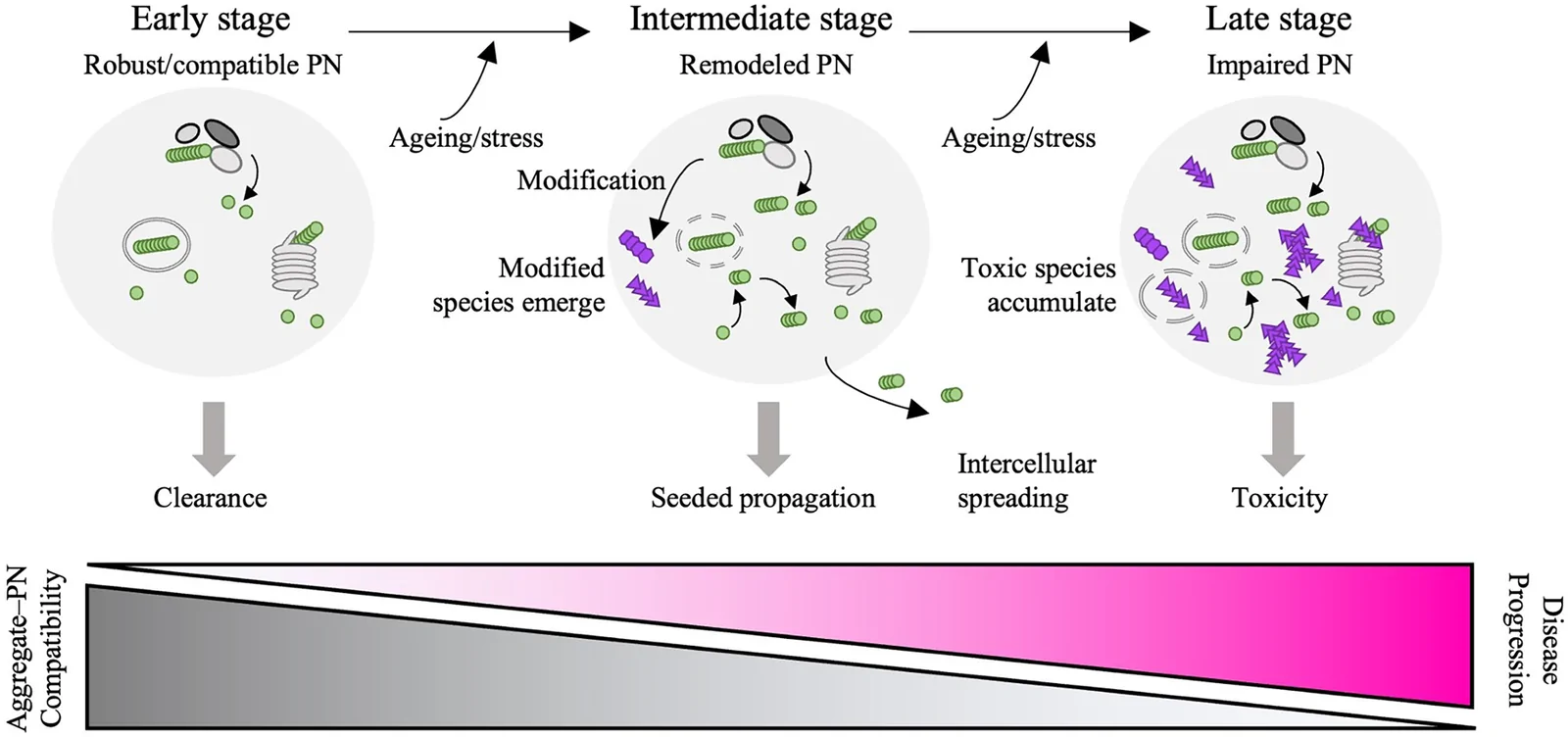
Co-evolution of aggregate identities and PN identities during disease progression. Conceptual model illustrating that aggregate identities and proteostasis network (PN) identities are dynamic and change during ageing and disease progression. In early stages, specific aggregates (green) are efficiently recognized, processed and cleared by a robust and compatible PN. During intermediate stages, PN identity is reshaped by factors such as ageing and stress, altering network composition and functional capacity. As a result, the same aggregates may now be handled differently, leading to incomplete processing, seeded propagation and intercellular spreading. Partial processing can also generate aggregate species with altered intrinsic properties, including truncated or covalently modified assemblies (purple aggregates). In later stages, further PN decline or impairment shifts aggregate handling again, promoting the accumulation of aggregate species with increased toxic potential, which can ultimately drive neurodegeneration. The gradient bar summarizes the progressive decline in aggregate–PN compatibility during disease progression, reflecting the increasing mismatch between evolving aggregate species and the changing functional capacity of the PN.

An important implication of this co-evolution is that aggregate propagation can be uncoupled from toxicity. As originally proposed in the prion field, efficient propagation of a fibrillar species does not necessarily coincide with neurotoxicity, which may instead arise from transient or low-abundance species generated during aggregate processing [8]. Within a changing PN environment, new aggregate species can emerge over time that differ in both seeding capacity and toxic potential (figure 2). This offers a mechanistic explanation for why high aggregate burden or robust spreading can coexist with limited toxicity in some contexts, while rapid neuronal loss occurs in others.

This perspective also has important consequences for disease modelling. Widely used patient-derived induced pluripotent stem cell models are effectively rejuvenated during reprogramming and therefore lack the ageing history that shapes proteostasis network identity *in vivo* [93]. As a result, key aspects of age-dependent PN decline and stress adaptation may be underestimated in these systems. Recent work using direct transdifferentiation approaches, which preserve age-associated cellular features, underscores the importance of cellular history for modelling neurodegenerative disease mechanisms more faithfully [93]. Complementary insights can also be obtained from ageing-competent model systems, such as *Caenorhabditis elegans* and *Drosophila*, in which proteostasis decline and stress history can be followed across the life course in a genetically tractable *in vivo* context [59,94–96].

Finally, this framework has important implications for therapeutic strategies. Interventions that aim to modulate proteostasis—either by targeting individual network components or by broadly enhancing proteostasis capacity—are likely to have context-dependent effects. The same intervention may be beneficial in one cellular or state context but ineffective or even harmful in another. A detailed understanding of aggregate–PN compatibility will therefore be essential for predicting therapeutic outcomes.

## Data Availability

This article has no additional data.

## Theme

One contribution of 20 to a Theo Murphy meeting issue ‘ProteostaSys: a systems view of proteostasis’.
